# The Relation of Tic Douloureux to Disorders of the Teeth

**Published:** 1852-07

**Authors:** Toogood Downing

**Affiliations:** Author of the Jacksonian Prize Essays of the Royal College of Surgeons, on Neuralgia, its various Forms, Pathology and Treatment.


### ARTICLE III.

*The Relation of Tic Douloureux to Disorders of the Teeth.* By TOOGOOD DOWNING, M. D. *Author of the Jacksonian Prize Essays of the Royal College of Surgeons, on Neuralgia, its various Forms, Pathology and Treatment.*

A FEW words of explanation may be advantageously premised, in order to start fair with the readers of a journal, who, we may presume, are chiefly of the dental profession, lest it be imagined that I am invading their province, and travelling into regions beyond my ken. *Ne sutor ultra crepidam*, I consider a very good axiom, and am, therefore, far from aspiring to be that unpopular Crispin who requires to be continually reminded, "that he should stick to his last."

For the dentists of the present day—I speak of course only of the best class—I entertain feelings of brotherly respect. They are not now mere tooth-drawers, or ignorant vendors of tooth powders and nostrums, as they were formerly styled with great show of reason ; but men of education and character, who bring to bear upon their department of the healing art, their specialty, the general principles of medicine and surgery. Some we know are excellent physiologists who take enlarged views of things, and aid, by researches in their particular department the onward progress of science. Observations, such as the following, may be addressed to them without fear of giving offence, as they are conceived in the spirit of kindness and good fellowship—certainly, with no wish to depreciate them in their own eyes, or in those of the public. The entire profession, in my opinion, should be bound together in the closest bonds of union. The links of the chain, such as where the different departments come in contact, are always the weakest ; and it is in order to strengthen these salient points that my present endeavor is directed.

My object is to draw particular attention to the sympathy subsisting between the nerves of the teeth, and those of the body in general, and the tendency of certain operations of the dentist to their disturbance; the derangement often caused in some conditions of health, by the mechanical appliances of artificial teeth, pivoting and stopping, and the injury accruing from the thoughtless and unnecessary extraction of sound and healthy members for the relief of painful conditions. The extent to which this latter practice is carried by, we may suppose, the more youthful and inexperienced members of the profession is almost incredible; and, therefore, we endeavor with candor to educe the laws which should regulate their proceedings in this particular. The efforts of art should be constantly directed rather to save than to destroy. Modern science fully recognises this principle. Sometime back, the surgeon made his boast and glory of the number of limbs he had amputated. Now, with more enlightened views, he justly prides himself upon the number he has saved. So should the dentist of the present day take credit, rather for the teeth he has preserved by judicious management, than for the hosts he has extracted by the most skillful and scientific wielding of the key or forceps In the course of my investigations into the nature and causes of neuralgic affections, this subject has been forcibly impressed upon my attention. I lay no claim to original views or discoveries in the matter. Many passing allusions to it are scattered through various writers, both medical and dental. I would wish merely to collect and reiterate these, and thus impress certain truths which are too often forgotten or disregarded.

Anatomy teaches us that the dental branches are supplied by the second and third divisions of the fifth pair of nerves, filaments of great tenuity, which enter at the fang and form the pulp in the interior. Upon their intimate organization, it is unnecessary to dwell, or their special purpose in the animal economy. They form part of the system of facial nerves, and are, therefore, subject to the same laws of subsistence and disturbance as those with which they are associated. On this point, there can scarcely be a difference of opinion. That they are of the sensitive kind most persons have been painfully reminded. It seems probable, that there is no admixture of motor fibrils in their composition, as is the case with other portions of the trifacial; but the presence of sympathetic filaments may be assumed both from the proximity of ganglia and the organic changes that take place; as a part of the nervous system of the face, the dental twigs are subject to certain painful disorders, which may either originate in themselves, or be conveyed to them from the adjoining branches. They may also arise from sympathy with distant organs or metastasis from them. This is familiar enough under the title of tooth-ache. But it is quite unnecessary for me to state that odontalgia is not the same disease under all circumstances. The character and nature of the pain varies in different instances as well as the pathological condition on which it depends. Thus, there may be inflammatory or rheumatic tooth-ache arising from inflammation or rheumatism of the odontoid nerves, and it is equally certain, that they may be afflicted with that agonizing disorder, known as tic-douloureux. As it is their relation to the latter malady, that I now more particularly wish to dwell upon, some of the characteristic features of the dis orders will be described, as I shall then be able to show the conditions under which the teeth become involved, either as a cause or effect of the complaint. Having so recently written largely on the subject, I should wish to compress my remarks into a very limited compass, as nothing is so "tedious as a twice told tale."

Neuralgia, as its name implies, is a painful affection of a nerve, consisting of a morbid exaltation of sensibility without organic change; a violent thrilling agony, compared by the sufferer to the shocks of an electric battery, occurring in paroxysms with more or less perfect intermission. It is not confined to any one part of the body, but may be seated in any portion of the system, wherever in fact the nerves are distributed. Thus it may occupy a position of the upper or lower extremity, or be placed in the head, chest or abdomen. Although, from causes which will be afterwards specified, its ravages are chiefly confined to the surface, yet physicians of the present day assign it a place among the affections of the internal organs, where it is equally characterised by violent intermittent suffering, which may continue for years without leaving a trace of organic disease after death.

Neuralgia, although sometimes erratic, is generally confined to distinct sets of nerves. Many of these forms, as they are called, have been distinguished by specific titles and regarded as distinct diseases. Such, for instance, as sciatica, where the chief nerve of the leg is implicated; hepatologia, or nephralgia, where those of the liver and kidney are affected, and tic douloureux when confined to the cheek. But the disorder is essentially the same wherever it is situated, however much the phenomena may be modified by local causes. These specific names are, therefore, retained more by the public than the sanction of the medical profession, as the object of all true science is to generalize, and such nomenclature is calculated to lead to erroneous notions of the pathology. When I say, however, that the disorder is essentially the same, I would wish to impose certain limitations to this expression. I distinguish three kinds, or varieties, of neuralgia—the spasmodic, the rheumatic, and the hysterical. Any nerve of the body may be affected with either of these disorders, but the painful derangement is of one or the other species.

It would be extremely interesting to know the exact nature of these nervous pains—the peculiar condition of the fibrils on which the impression depends. The mind of man is inquisitive; some portion of that which is considered woman's foible enters into his composition. He finds a satisfaction, a glow of pleasure in unravelling the secret mysteries of nature, as expressed by the poet—*Felix qui potint rerum cognoscere causes.*

In cases like the present, this propensity may be rationally and advantageously indulged. The talents and industry of man can never be better employed than in seeking to discover the sources of the trouble to which his species is condemned, and the only regret is, that in his most legitimate pursuits, he should so often be disappointed. Nothing positive is known on the subject. The physiology of the nervous system is very imperfectly understood, and hence its pathology can scarcely be satisfactorily elucidated. If we know not the nature and nerve of action of the *vis nervosi*—while it is still undetermined whether it be a circulating fluid—a refinement of galvanic electricity coming through its fibres in the same way as blood traverses the veins and arteries—or a vibratile or molicular motion of its organic elements, we must remain in the dark as to its derangements and disturbances. Researches by the scalpel afford no clue except by their negative nature. It is true that certain morbid appearances, such as hardening, or softening, thickening, or atrophy, of the nerves affected with neuralgia, have been sometimes discovered after death ; but these are opposed by the numerous examples where the tic douloureux had existed for a length of time without leaving the slightest trace of organic change. The most careful examinations by excellent anatomists have failed in elucidating anything abnormal. The nerve affected has been apparently in as sound condition as its fellow of the opposite side. Hence we may infer that hyperæmia, anæmia, &c., are not essential to the disorder, but are mere accessories to it, either before or after the fact. If organic changes were essential, it would be invariably present. Hence we may discard the theories of neuralgia depending upon inflammation, or acrid matter, cancer, &c., and view the derangement as entirely functional. Regarded in this light the notion of Dr. Maünlloch, that neuralgia is an intermittent fever of the part—a kind of local ague, in fact—appears extremely plausible; more especially when its frequent origin is malaria, its association with ague, and its periodicity are taken into consideration. But there are serious objections to regarding it as always of this nature, and we are hence driven to further hypothesis and conjecture.

My own views on the subject are peculiar. From an attentive observation of the phenomena, and by a train of reasoning which it is here impossible to follow, I have arrived at the conclusion that neuralgia is often a local disease, depending entirely on topical causes; that it is entirely functional in character, and that this function is so much exalted by the nervous excitant as to give rise to *spasm* of the nervous fibril affected. The suddenness of recession and of remission; the perfect interruption of the paroxysms generally, and the peculiar sensations of the patient, all tend to this conclusion. This, of course does not preclude the idea of the disorder having a frequent origin in some general or centric derangement, such as is shown by its occasionally invading a variety of parts in succession and flying about from one region to another. This demonstrates the existence of a particular state of the system, or disease of the blood, perhaps, when the nerves take on this abnormal irritability. But on what this neuralgic *diathesis* absolutely depends, we have no means at present to determine.

(To be Continued.)

